# Factors Associated with Frailty in Patients with Active Inflammatory Bowel Disease: A Single-Center Observational Study

**DOI:** 10.3390/medicina62061194

**Published:** 2026-06-21

**Authors:** Mihaela Topala, Victor Ionescu, Ioana Gabriela Lupescu, Gabriel Becheanu, Cristian Gheorghe

**Affiliations:** 1 Doctoral School, Carol Davila University of Medicine and Pharmacy, 020021 Bucharest, Romania; 2 Center of Gastroenterology and Hepatology, Fundeni Clinical Institute, 022328 Bucharest, Romania; drcgheorghe@gmail.com; 3 Infection Control Unit, Bucharest Clinical Emergency Hospital, 014461 Bucharest, Romania; v.stefan@ymail.com; 4 Center for Excellence in Translational Medicine, Fundeni Clinical Institute, 022328 Bucharest, Romania; 5 Department of Radiology, Medical Imaging and Interventional Radiology I, Carol Davila University of Medicine and Pharmacy, 020021 Bucharest, Romania; ioana.lupescu@umfcd.ro; 6 Department of Radiology and Medical Imaging, Fundeni Clinical Institute, 022328 Bucharest, Romania; 7 Department of Pathology, Carol Davila University of Medicine and Pharmacy, 020021 Bucharest, Romania; gabriel.becheanu@umfcd.ro; 8 Department of Pathology, Fundeni Clinical Institute, 022328 Bucharest, Romania; 9 Discipline of Gastroenterology and Hepatology, Department of Internal Medicine, Carol Davila University of Medicine and Pharmacy, 020021 Bucharest, Romania

**Keywords:** inflammatory bowel disease, Crohn’s disease, ulcerative colitis, frailty, sarcopenia, malnutrition

## Abstract

*Background and Objectives:* Frailty is increasingly recognized as a clinically relevant condition in inflammatory bowel disease (IBD), yet available data remain limited and are often derived from indirect assessment methods. This study aimed to assess frailty using a Fried-derived index and to identify factors associated with frailty in adults with active IBD. *Materials and Methods:* We analyzed baseline data from a prospective, single-center cohort of adult patients hospitalized with active IBD who required initiation of biologic therapy or small molecules. Frailty was assessed using a modified Fried frailty index comprising shrinking, exhaustion, weakness, slowness, and low physical activity. Patients fulfilling ≥3 criteria were classified as frail, while those meeting 1–2 criteria were classified as pre-frail. Clinical, anthropometric, functional, laboratory, and questionnaire-based patient-reported measures were compared between frail and non-frail patients. Multivariate logistic regression was used to identify factors independently associated with frailty. *Results:* Seventy-five patients were included, of whom 45 (60%) were male and 44 (58.7%) had ulcerative colitis. The median age was 37 years. Frailty was identified in 17 (22.7%) patients, and 47 (62.7%) were pre-frail. Frail patients were younger (*p* = 0.001) and had a shorter disease duration (*p* = 0.036), higher disease activity scores (*p* = 0.005), higher C-reactive protein levels (*p* = 0.006), lower hemoglobin levels (*p* = 0.030) and serum albumin concentrations (*p* < 0.001), and lower body mass index (*p* < 0.001). In multivariate analysis, shorter disease duration (OR = 0.83, 95% CI: 0.70–0.97, *p* = 0.021) and lower serum albumin levels (OR = 0.17, 95% CI: 0.05–0.59, *p* = 0.005) were independently associated with frailty, whereas severe disease (OR = 2.14, 95% CI: 0.52–8.86, *p* = 0.294) was not significant after adjustment. *Conclusions:* Frailty and pre-frailty were highly prevalent in this selected cohort of relatively young adults with active IBD. Shorter disease duration and lower albumin levels were independently associated with frailty. Assessing frailty status may help identify vulnerable patients who could benefit from closer monitoring and multidisciplinary interventions.

## 1. Introduction

The incidence of inflammatory bowel disease (IBD), mainly represented by Crohn’s disease (CD) and ulcerative colitis (UC), has increased in recent decades, particularly in newly developed industrial regions. In 2021, 3.8 million people worldwide were living with IBD, of whom almost 10% were newly diagnosed. The global burden of IBD is particularly increasing among pediatric and elderly populations, which are vulnerable and challenging to manage [1].

Frailty represents a clinical syndrome characterized by physical and psychological impairment, and, consequently, by a state vulnerable to stressors [2], that was initially described in the elderly population. In 2001, Fried et al. conceptualized frailty in a cohort of older adults as an index that encompassed five aspects—weight loss, weakness, exhaustion, slowness, and low physical activity [3]. The mechanisms of frailty are assumed to arise from modifications in the immune system and from the proinflammatory state that occurs during the ageing process—the “inflammageing” theory [4]. Over the years, numerous tools that assess frailty have been developed [5,6,7,8], and, moreover, its assessment has been extended to patients with chronic diseases or undergoing surgical interventions [9].

Frailty evaluation and its impact on the outcome of patients with IBD have been an emerging subject during the last years. In two of the landmark studies that prompted interest in this topic, Kochar et al. reported that frail patients had a twofold higher risk of infections after starting immunosuppressive treatment [10] and almost threefold higher odds of mortality [11], independently of potential confounders, including age. Frailty was further linked with the disease outcome, being associated with morbidity, mortality, hospitalizations and postoperative complications in an IBD population [12].

Considering the limited number of studies published on this matter, frailty in IBD remains an underdeveloped topic in the literature. Most available data are derived from indirect methods of frailty assessments, such as scores based on multimorbidity or diagnoses of frailty-related domains. Some studies reported information from elderly IBD populations exclusively. Therefore, our aim was to evaluate frailty through a direct method, using a Fried-derived index, and to identify factors associated with frailty in an adult population of patients with active IBD.

## 2. Materials and Methods

### 2.1. Data and Patient Population

We report the baseline data from a prospective, single-center, cohort study conducted in Fundeni Clinical Institute. The present study represents a cross-sectional analysis of baseline data from this prospective cohort; therefore, temporal and causal relationships cannot be established. Ethical approval was obtained from the Ethics Committee of Fundeni Clinical Institute (No. 46272, 8 September 2020), and the study was conducted in accordance with the principles of the Declaration of Helsinki, as revised in 2013. All participants provided their written informed consent. We enrolled patients with a histopathologically confirmed diagnosis of IBD, admitted in the Gastroenterology Department during the periods January 2021–March 2022 and October 2023–August 2025, with active disease that required biological or small molecule initiation. Disease activity was assessed using the Crohn’s Disease Activity Index (CDAI) in patients with CD and the full Mayo score in patients with UC. During the same hospitalization, patients underwent a comprehensive assessment that included colonoscopy, laboratory and clinical evaluation, and imaging, when clinically indicated. In CD, endoscopic and imaging findings were considered as part of the overall clinical evaluation and were not analyzed separately in relation to frailty. The exclusion criteria were age under 18 years old, major comorbidities that could impair the physical and psychological status of the patient (e.g., heart failure, ischemic heart disease, diabetes mellitus, chronic kidney disease, chronic obstructive pulmonary disease, chronic liver disease, hematologic disorders, neurological disorders, severe psychiatric disorders, or malignancy), pregnancy or lactation, and refusal or inability to provide written informed consent.

Anthropometric measurements were performed according to the National Health and Nutrition Examination Survey (NHANES) Anthropometry Procedures Manual [13]. Measurements were repeated three times, and the mean value is reported. Frailty was assessed using a modified Fried frailty phenotype, comprising five domains: shrinking, exhaustion, weakness, slowness, and low physical activity [3]. Shrinking was defined as unintentional weight loss ≥ 4.5 kg or ≥5% of body weight during the preceding 12 months and was assessed by patient self-report. The duration of the reported weight loss was also recorded. Exhaustion was self-assessed through two questions (“I felt that everything I did was an effort” and “I could not get «going»”) from the Center for Epidemiologic Studies Depression Scale (CES-D). The first two domains replicate the Fried phenotype. Weakness was defined as handgrip strength (HGS) below the age- and sex-specific 20th percentile of normative reference values published in the Jamar^®^ manual, based on data reported by Mathiowetz et al. [14]. Because the normative data were available as the mean and standard deviation, the 20th percentile was approximated assuming normality as mean − 0.842 × SD. The thresholds applied are provided in Supplementary Table S1. Slowness was defined as a gait speed < 0.8 m/s during the 4.5 m walk test [15]. Low physical activity was defined as the “Low” category of the International Physical Activity Questionnaire (IPAQ). Each domain was scored as present or absent. Frailty was defined as the presence of ≥3 criteria, pre-frailty as 1–2 criteria, and robustness as no criteria, consistently with the original definition. The operational definitions used for each frailty domain are summarized in Table 1.

HGS was measured using a Jamar^®^ Hydraulic Dynamometer (Lafayette Instrument Company, Lafayette, IN, USA) following the standardized protocol recommended by the American Society of Hand Therapists [16]. Participants were seated on a chair with both feet on the floor, the elbow flexed at 90°, and the wrist slightly extended. While holding the device, the proximal interphalangeal joint of the ring finger was positioned at a 90° angle, with the thumb and fingers aligned on the same side of the device. Three measurements were performed for each hand, and the mean value for each hand was recorded. For the frailty score, weakness was defined using the mean HGS of the dominant hand; values below the age- and sex- specific 20th percentile of the Mathiowetz normative data were classified as weakness.

Age- and sex-adjusted HGS Z-scores were calculated as a sensitivity analysis. The European Working Group on Sarcopenia in Older People guideline (EWGSOP2) HGS thresholds were used to identify probable sarcopenia. The chair stand test was evaluated as an additional descriptive measure of low muscle strength. The 400 m walk test was used to assess physical performance. These secondary assessments and their corresponding cut-offs are summarized in Table 2. Considering that validated thresholds for these measures are lacking in younger populations, the EWGSOP2 cut-offs were applied and should be interpreted with caution [15]. Except for the 400 m walk test, all measurements were repeated three times, and the mean was calculated. Mid-arm muscle circumference (MAMC) was calculated using the formula mid-upper arm circumference (MUAC)—π × triceps skinfold thickness (TSF).

Body mass index (BMI) was calculated as weight (kg) divided by height (meters) squared. World Health Organization categories were used to classify BMI as underweight (<18.5 kg/m^2^), normal (18.5–24.9 kg/m^2^), overweight (25.0–29.9 kg/m^2^), obese class I (30.0–34.9 kg/m^2^), obese class II (35.0–39.9 kg/m^2^), or obese class III (≥40.0 kg/m^2^). Underweight was further categorized into mild thinness (17.0–18.5 kg/m^2^), moderate thinness (16.0–17.0 kg/m^2^), or severe thinness (<16.0 kg/m^2^) [17].

### 2.2. Statistical Analyses

Continuous variables are presented as the mean ± standard deviation or median (interquartile range), depending on their distribution. Categorical variables are presented as counts and percentages. Data distributions were assessed using the Shapiro–Wilk test [18,19]. Comparisons between frail and non-frail patients were performed using the independent samples *t*-test for normally distributed continuous variables and the Mann–Whitney U test for non-normally distributed variables. Categorical variables were compared using the chi-square test or Fisher’s exact test, as appropriate. HGS values were correlated to the normative grip strength chart, and an age- and sex-adjusted Z-score was calculated. Univariate and multivariate logistic regression analyses were conducted. Variables included in the multivariate model were selected based on their clinical relevance and the univariate analysis (*p* < 0.10). Variables that constituted components of the frailty definition were excluded. Because of the limited number of frailty events, the multivariate model was restricted to three predictors to reduce overfitting and improve estimate stability. All statistical tests were two-sided, and a *p*-value < 0.05 was considered statistically significant. Statistical analyses were performed using IBM^®^ SPSS^®^ Statistics version 23.0.0.0 software.

## 3. Results

Seventy-five patients were included, out of which 45 (60.0%) were males and 44 (58.7%) were diagnosed with UC. The median age was 37.0 (IQR, 28.0–44.0) years old, and the median disease duration was 4.5 (IQR, 1.0–10.2) years. Only five patients were older than 60 years, none of them meeting the criteria for frailty, although three were pre-frail. The median CDAI score in the CD subgroup was 220 (IQR, 156–308), whereas the median full Mayo score in the UC subgroup was 11 (IQR, 9–11). Ten patients (13.3%) had extraintestinal manifestations—all had articular involvement, three also had dermatological lesions and two had ocular manifestations.

At the time of evaluation, 43 patients (57.3%) were receiving corticosteroids, 36 (48.0%) were receiving 5-aminosalicylates, 21 (28.0%) had previously received biologic or small molecule treatment, and 16 (21.3%) were taking immunomodulators, without a significant difference between the frail and non-frail subgroups. Among patients previously exposed to biologic therapy or small molecules, 12 (16.0% of the total cohort) had received one treatment line, seven (9.3%) had received two lines, and two (2.7%) had received three lines.

Within the preceding 12-month assessment window, 58 of 75 (77.3%) patients reported unintentional weight loss, with a median loss of 6 (IQR, 2–12) kg, occurring over a median period of 3 (IQR, 2–6) months. The mean BMI of the 75 patients was 22.3 ± 4.1 kg/m^2^. Forty (53.3%) patients had a normal BMI, 16 (21.3%) were overweight, four (5.3%) had class I obesity, and 15 (20.0%) were underweight. Among the underweight patients, eight (10.7% of the total cohort) had mild thinness, two (2.7%) had moderate thinness, and five (6.7%) had severe thinness.

Muscle strength assessed using the five-repetition chair stand test showed no significant difference between frail and non-frail patients. Weakness was assessed using three approaches: the primary Fried-derived definition incorporated into the frailty score, a Z-score-based sensitivity analysis, and the EWGSOP2 cut-offs used for the descriptive assessment of probable sarcopenia. Weakness based on the Fried-derived definition was highly prevalent, affecting 51 (68.0%) of the patients. A similar prevalence was observed using a Z-score threshold of ≤−1, which identified weakness in 48 (64.0%) patients, while severe weakness, defined as a Z-score ≤ −2, was present in 13 (17.3%) patients. Probable sarcopenia according to the EWGSOP2 HGS cut-offs was identified in six (8.0%) patients. These findings suggest that functional impairment was substantially more common than probable sarcopenia-level muscle weakness in the patients with active IBD. During the assessment of physical performance using the 400 m walk test, one patient declined to perform the test; two patients discontinued it because of joint pain and an urgent need for a bowel movement, respectively; and five patients (6.5%) were unable to complete the test because of weakness.

Seventeen patients were frail, 47 were pre-frail, and 11 were robust. The prevalence of each individual frailty domain is presented in Table 2, while the final frailty classification of the cohort is summarized in Table 3.

Frail patients were significantly younger than non-frail patients (median, 28.0 vs. 39.5 years, *p* = 0.001) and had a shorter disease duration (*p* = 0.036). Disease activity scores were higher in frail patients, including the CDAI (*p* = 0.003) and full Mayo score (*p* = 0.008). As expected from the frailty criteria, descriptive comparisons showed lower HGS (*p* = 0.007) and lower HGS Z-scores (*p* < 0.001), higher CES-D scores (*p* < 0.001), and lower IPAQ physical activity levels (*p* < 0.001) in frail individuals. Frail patients also had higher inflammatory marker levels (C-reactive protein, *p* = 0.006), lower hemoglobin levels (*p* = 0.030), lower albumin levels (*p* < 0.001), lower BMI (*p* < 0.001), and greater weight loss (*p* < 0.001). No significant differences were observed in terms of sex, disease location, or most treatment variables. The baseline characteristics of patients with frail and non-frail phenotypes are described in Table 4.

In the multivariate logistic regression analysis, shorter disease duration (OR = 0.83, 95% CI: 0.70–0.97; *p* = 0.021) and lower albumin levels (OR = 0.17, 95% CI: 0.05–0.59; *p* = 0.005) were independently associated with frailty. Severe disease was not significantly associated with frailty after adjustment (OR = 2.14, 95% CI: 0.52–8.86; *p* = 0.294) (Table 5).

## 4. Discussion

This study assessed the presence of frailty in patients with active IBD using a modified Fried-derived index and identified factors associated with frailty in this patient population. Approximately one-quarter of the patients were frail, and almost two-thirds were pre-frail. After variables that constituted or directly overlapped with the frailty definition were excluded, several other factors were associated with frailty, including younger age, shorter disease duration, higher CRP, lower albumin levels, and severe disease. In a multivariate analysis, only shorter disease duration and lower albumin levels remained independently associated with frailty. Severe disease may have lost its significance because of its overlap with serum albumin, as both variables capture aspects of inflammatory disease burden. Given the few frailty events, this finding warrants cautious interpretation.

The prevalence of frailty and pre-frailty in our cohort was comparable to the prevalence reported in the literature. A meta-analysis comprising nearly 1.9 million adults with IBD showed a pooled prevalence of 18%, with approximately one-third of elderly patients with IBD classified as frail [12]. The prevalence of pre-frailty varies between 28.0 and 59.4% [20,21,22,23]. The slightly higher rates of frailty in our cohort could be explained by the inclusion of only patients with active disease, who are susceptible to weight loss, malnutrition, impaired quality of life, and sarcopenia. The high prevalence of weight loss and weakness in our study likely reflects the active disease status of the cohort and supports the burden of physical frailty-related impairment in active IBD. The distribution of individual frailty domains further supports this interpretation, as shrinking and weakness were the predominant components, whereas exhaustion, low physical activity, and slowness were less frequent. Weight loss, weakness and exhaustion represent mechanisms contributing to disease-associated frailty rather than separate confounding symptoms. Because HGS thresholds depend on the normative references and measurement protocol, a stricter Z-score sensitivity analysis was performed. The consistency between the percentile-based (68.0% of patients) and Z-score-based (64.0% of patients) definitions supports the robustness of the high prevalence of weakness in patients with active IBD. This weakness might partly reflect nutritional impairment, given the lower BMI and albumin levels and greater weight loss observed in frail patients.

Interestingly, in our study, younger age and shorter disease duration were associated with frailty. We excluded patients with major comorbidities that could confound the IBD-related frailty; many such conditions are more common in older adults. Therefore, only five patients were older than 60 years old, none of whom were frail, although three were pre-frail. The frailty observed in this cohort may therefore predominantly reflect the inflammatory, nutritional, and functional burden of active IBD rather than age-related or multimorbidity-related frailty. Nevertheless, the associations with younger age and shorter disease duration may also reflect the selected cohort and should be interpreted cautiously. Younger patients with newly diagnosed disease might have experienced a prolonged symptomatic period before diagnosis, potentially increasing their susceptibility to frailty compared with older patients with a longer established disease course. Patients with a longer disease duration might also have been under regular monitoring, allowing flares to be identified and managed more promptly and the burden of their systemic effects to be limited. However, this assumption should not be extrapolated to the broader older IBD population, particularly patients with comorbidities. Boyd et al. reported that older patients with acute severe UC were less likely to receive medical salvage therapy, particularly when frailty was present, and were more likely to undergo urgent or emergent surgery than younger patients. The elderly group also experienced high mortality rates, approaching 30% within one year of admission [24]. Moreover, by analyzing a cohort of 405 older patients with IBD, Asscher et al. identified a high prevalence of geriatric deficits associated with disease activity and disease burden. Compared with patients with non-elderly-onset IBD, those with elderly-onset IBD had higher rates of cognitive impairment, lower HGS values, and slower gait speed, even when the disease was biochemically inactive [25].

The reported frailty prevalence varies according to the assessment method. Commonly used tools include scores based on administrative claims, such as International Classification of Diseases diagnoses, and indices derived from multimorbidity. Studies using multimorbidity-based frailty indices in patients undergoing intestinal resection have reported low prevalence estimates of 2.5–4.7% [26,27,28]. These results warrant cautious interpretation, since key components of physical frailty were not directly assessed. Thus, younger patients without comorbidities but with IBD-related frailty might not have been taken into consideration.

Factors associated with frailty have differed across studies. In an Italian cohort of 386 patients with IBD assessed using the original Fried frailty phenotype, frailty was associated with female sex, active disease, and current use of corticosteroids; however, only active disease remained independently associated with frailty [20]. In a Chinese cohort, BMI, elevated erythrocyte sedimentation rate, sleep impairment, and depression were correlated with frailty [21]. Another Chinese study identified older age, longer IBD duration, disease activity, sleep impairment, elevated inflammatory markers, and low serum albumin as risk factors for frailty [29]. Liu et al. similarly reported associations between frailty and lower BMI, malnutrition, active disease, and low serum albumin [23]. We also observed an association between the serum albumin level and frailty. Beyond its role as a marker of nutritional status, serum albumin is influenced by systemic inflammation and might reflect disease activity, particularly in severe cases. Serum albumin also appears to have prognostic value and may help guide treatment decisions and monitor disease activity [30].

Previous studies reported that frailty in IBD was associated with higher rates of readmission, severe IBD-related hospitalization, mortality [11,31,32], infection after immunosuppression [10], and postoperative morbidity and mortality [26,28]. Thus, frailty represents a predictive factor for negative outcomes in IBD patients, and by its integration into clinical practice, it could contribute to early risk assessment and tailored management. Frailty screening may be particularly relevant in patients with active IBD, especially at diagnosis, during severe flares or hospitalizations, during preoperative assessment or before treatment escalation. Since frailty captures early functional decline, implementing rehabilitation measures might improve the disease course and lower the risk of complications. Patients identified as frail or pre-frail may benefit from nutritional assessment, physical rehabilitation, and closer multidisciplinary monitoring. Future studies should focus on clarifying the underlying pathways of frailty progression, identifying feasible screening methods, assessing the possibility of frailty reversal, and designing targeted interventions for everyday practice.

Most methods currently used to assess frailty were developed in elderly cohorts. This might limit their generalizability in IBD populations, since IBD-specific factors are not taken into consideration. Moreover, direct methods of evaluation, including the Fried frailty index or Fried-derived indices, although accurate, can be cumbersome to apply in clinical practice and difficult to implement in large cohorts. Readily applicable tools tailored to the IBD population still need to be validated. Recently, a study group from Italy reported the development and validation of an IBD-specific tool—the IBD Frailty Score, an easy-to-implement scoring system of 28 items—which proved to have acceptable accuracy for distinguishing frail from fit patients when compared with the Fried frailty phenotype [33]. However, further studies are needed to establish its role in frailty assessment, particularly among patients with moderate-to-severe active disease.

The strength of our study is represented by the prospective observational design, which allowed for standardized data collection. We adapted Fried phenotype criteria according to sex and age and implemented a reliable bedside method to measure frailty. Moreover, by excluding patients with multimorbidity, we aimed to reduce the confounding factors that could interfere with the results.

This study has several limitations. First, the cohort was small and highly selected, comprising only adults with active IBD requiring initiation of advanced therapy at a single tertiary center. This selection reflected our specific aim of evaluating frailty in patients with clinically significant active disease, but it limits the external validity of the findings. Therefore, the results should not be generalized to the overall IBD population, particularly older adults with multimorbidity or patients with mild or quiescent disease who do not require advanced therapy initiation. Second, because the analysis was cross-sectional at baseline, temporal relationships and causality cannot be established; the identified factors should be interpreted as associations with frailty rather than causes or predictors. Third, although we used a clinically meaningful direct frailty assessment, the phenotype was modified from the original Fried index, and some component thresholds have not been specifically validated in younger IBD populations. Finally, the small number of frailty events limited the statistical power of the multivariate analysis. Although the model was restricted to three predictors to reduce the risk of overfitting, the estimates may remain imprecise or unstable, and moderate associations may not have been detected. Therefore, the non-significant findings should be interpreted cautiously, and the results should be considered exploratory and requiring validation in larger cohorts.

## 5. Conclusions

Frailty and pre-frailty were highly prevalent among patients with active inflammatory bowel disease, even in this relatively young population, when age-adjusted definitions were used. In this context, the observed phenotype likely reflects the inflammatory, nutritional, and functional burden of active disease rather than geriatric or multimorbidity-related frailty. Shorter disease duration and lower albumin levels were independently associated with frailty, whereas severe disease was not significant after adjustment. These findings support further evaluation of frailty screening in patients with active inflammatory bowel disease requiring advanced therapy, while larger and more representative studies are needed before broader implementation. Further studies are needed to develop and validate easily applicable tools for frailty assessment and to investigate potential therapeutic interventions.

## Figures and Tables

**Table 1:** Secondary functional and sarcopenia-related assessments.

Assessment	Test or Variable	Cut-Off or Definition	Role in the Analysis
HGS Z-score	Age- and sex-adjusted HGS Z-score	Z-score ≤ −1: weakness Z-score ≤ −2: severe weakness	Sensitivity analysis
EWGSOP2 handgrip strength	Mean dominant-hand HGS	<27 kg for men; <16 kg for women	Low-muscle-strength criterion; used to identify probable sarcopenia
Chair stand test	Time required to complete five chair rises	>15 s	Alternative low-muscle-strength criterion
400 m walk test	Time to complete the 400 m walk test	>6 min or inability to complete the test	Physical performance measure

Abbreviations: EWGSOP2—European Working Group on Sarcopenia in Older People 2; HGS—handgrip strength.

**Table 2:** Operational definitions and prevalence of the individual frailty domains in patients with active inflammatory bowel disease.

Frailty Domain	Operational Definition	Number of Patients, n/N (%)
Shrinking	Unintentional weight loss of ≥4.5 kg or ≥5% of body weight occurring within the preceding 12 months. Weight loss was assessed by patient self-report.	53/75 (70.7%)
Exhaustion	Exhaustion assessed using the two CES-D items “I felt that everything I did was an effort” and “I could not get going,” according to the predefined response threshold used in the Fried phenotype—criterion is met if patient answers “moderate amount of the time” or “most of the time” to either one or both questions.	18/75 (24.0%)
Weakness	Mean dominant-hand HGS below the age-, sex-, and hand-specific 20th percentile derived from the Mathiowetz normative values. The thresholds used are presented in Supplementary Table S1.	51/75 (68.0%)
Slowness	Gait speed < 0.8 m/s during the 4.5 m walk test.	4/75 (5.3%)
Low physical activity	Physical activity classified as “Low” according to the IPAQ categories.	10/75 (13.3%)

Abbreviations: CES-D—Center for Epidemiologic Studies Depression Scale; HGS—handgrip strength; IPAQ—International Physical Activity Questionnaire.

**Table 3:** Final frailty classification of the study cohort.

Frailty Classification	Number of Frailty Criteria	Patients, n/N (%)
Robust	0	11/75 (14.7%)
Pre-frail	1–2	47/75 (62.7%)
Frail	≥3	17/75 (22.7%)

**Table 4:** Baseline characteristics of frail versus non-frail patients with active inflammatory bowel disease.

Variable	Frail Group (17 Patients)	Non-Frail Group (58 Patients)	*p* Value
Age (years), median (IQR)	28.0 (22.0–35.0)	39.5 (29.7–52.2)	0.001
Male sex, n (%)	8 (47.1%)	37 (63.8%)	0.216
Disease duration (years), median (IQR)	1.0 (0.0–5.5)	6.0 (1.0–11.0)	0.036
Crohn’s disease, n (%)	5 (29.4%)	26 (44.8%)	0.256
Age at diagnosis			
≤16 years, n/N (%)	1/5 (20.0%)	1/26 (3.8%)	0.301
17–40 years, n/N (%)	4/5 (80.0%)	18/26 (69.2%)	1.000
>40 years, n/N (%)	0/5 (0.0%)	7/26 (26.9%)	0.562
Disease location			
Terminal ileum, n/N (%)	1/5 (20.0%)	7/26 (26.9%)	1.000
Colonic, n/N (%)	2/5 (40.0%)	4/26 (15.4%)	0.241
Ileocolonic, n/N (%)	2/5 (40.0%)	15/26 (57.7%)	0.636
Disease behavior			
Inflammatory, n/N (%)	0/5 (0.0%)	10/26 (38.5%)	0.147
Stricturing, n/N (%)	3/5 (60.0%)	11/26 (42.3%)	0.636
Penetrating, n/N (%)	2/5 (40.0%)	5/26 (19.2%)	0.562
Perianal lesions, n/N (%)	2/5 (40.0%)	7/26 (26.9%)	0.613
Ulcerative colitis, n (%)	12 (70.6%)	32 (55.2%)	0.256
Left-sided, n/N (%)	2/12 (16.7%)	12/32 (37.5%)	0.282
Extensive, n/N (%)	10/12 (83.3%)	20/32 (62.5%)	0.282
Extraintestinal manifestations, n (%)	2 (11.8%)	8 (13.8%)	1.000
BMI (kg/m^2^), mean ± SD	19.2 ± 3.6	23.3 ± 3.8	<0.001
Prior IBD related-surgery, n (%)	4 (23.5%)	4 (6.9%)	0.072
Disease severity			
Severe disease, n (%)	12 (70.6%)	19 (32.8%)	0.005
CDAI score (points), median (IQR)	453.0 (309.0–482.0)	198.5 (153.5–282.5)	0.003
Mild CD, n (%)	0 (0.0%)	13 (22.4%)	0.032
Moderate CD, n (%)	2 (11.8%)	10 (17.2%)	0.723
Severe CD, n (%)	3 (17.6%)	3 (5.2%)	0.126
Mayo score (points), median (IQR)	11.5 (10.2–12.0)	10.5 (8.0–11.0)	0.008
Moderate UC, n (%)	3 (17.6%)	16 (27.6%)	0.534
Severe UC, n (%)	9 (52.9%)	16 (27.6%)	0.051
Laboratory tests			
C-reactive protein (mg/L), median (IQR)	33.6 (11.2–72.9)	11.1 (3.7–26.8)	0.006
Fibrinogen (mg/dL), mean ± SD	516.7 ± 128.5	477.5 ± 129.8	0.281
Hemoglobin (g/dL), mean ± SD	11.8 ± 1.8	13.0 ± 2.1	0.030
White blood cells (×10^3^/μL), median (IQR)	9.7 (5.7–11.6)	8.3 (7.0–9.9)	0.830
Thrombocytes (×10^3^/μL), median (IQR)	361.0 (316.0–451.0)	343.0 (287.0–461.0)	0.285
Albumin (g/dL), mean ± SD	3.5 ± 0.6	4.2 ± 0.6	<0.001
Ferritin (ng/mL), median (IQR)	60.9 (37.0–397.0)	46.2 (10.3–85.0)	0.094
Fecal calprotectin (mcg/g), median (IQR)	1500.0 (953.0–4364.0)	1000.0 (263.0–2258.7)	0.270
Treatment			
5-Aminosalicylates, n (%)	7 (41.2%)	29 (50.0%)	0.522
Corticosteroids, n (%)	12 (70.6%)	31 (53.4%)	0.209
Immunomodulators, n (%)	1 (5.9%)	15 (25.9%)	0.099
Biologics, n (%)	5 (29.4%)	16 (27.6%)	1.000
Weight loss (kg), median (IQR)	11.0 (6.2–14.7)	7.0 (4.0–12.0)	<0.001
Period of weight loss (months), median (IQR)	3.5 (1.2–6.0)	3 (2.0–5.2)	0.945
4.5 m test (seconds), median (IQR)	4.2 (3.3–4.4)	3.6 (3.3–4.1)	0.055
400 m test (seconds), median (IQR)	321.5 (293.5–340.0)	302.0 (272.5–327.2)	0.160
Chair stand test (seconds), mean ± SD	13.6 ± 5.2	12.4 ± 2.9	0.413
Handgrip strength (kgf), mean ±SD	26.4 ± 11.2	35.4 ± 10.8	0.007
Waist (cm), mean ± SD	74.1 ± 9.4	85.4 ± 11.0	<0.001
Mid-arm muscle circumference (cm), median (IQR)	18.6 (16.1–21.5)	20.9 (17.3–22.6)	0.086
Z-score, median (IQR)	−1.6 (−2.5 to −1.4)	−1.2 (−1.6 to −0.5)	<0.001
SARC-F (points), median (IQR)	2.0 (0.2–2.7)	0 (0.0–1.0)	<0.001
CES-D (points), median (IQR)	26.5 (18.0–34.0)	8.5 (3.0–13.0)	<0.001
IPAQ (MET-min/week), median (IQR)	1974.5 (563.6–4091)	4285.5 (2552.6–7249.5)	<0.001
Daily sedentary time (minutes), median (IQR)	420.0 (255.0–487.0)	300.0 (180.0–532.0)	0.164

Abbreviations: BMI—body mass index; CES-D—Center for Epidemiologic Studies—Depression Scale; CDAI—Crohn’s Disease Activity Index; IPAQ—International Physical Activity Questionnaire; IQR—interquartile range; SD—standard deviation.

**Table 5:** Results of univariate and multivariate logistic regression of factors associated with frailty in patients with active inflammatory bowel disease.

	Univariate Analysis		Multivariate Analysis	
Predictor	Odds Ratio (95% Confidence Interval)	*p* Value	Odds Ratio (95% Confidence Interval)	*p* Value
Age	0.90 (0.85–0.96)	0.003		
Male sex	0.50 (0.17–1.50)	0.220		
Disease duration	0.91 (0.82–1.01)	0.087	0.83 (0.70–0.97)	0.021
Crohn’s disease	0.51 (0.16–1.64)	0.261		
Extraintestinal manifestations	0.83 (0.16–4.35)	0.829		
Body mass index	0.73 (0.60–0.88)	0.001		
Severe disease	4.90 (1.51–16.01)	0.008	2.14 (0.52–8.86)	0.294
C-reactive protein	1.01 (1.00–1.02)	0.014		
Albumin level	0.22 (0.09–0.56)	0.001	0.17 (0.05–0.59)	0.005
Hemoglobin	0.74 (0.56–0.99)	0.043		
Corticosteroids	2.09 (0.65–6.69)	0.214		

## Data Availability

Data used for the statistical analyses can be shared on request to the corresponding author.

## Supplementary Materials

The following supporting information can be downloaded at: https://www.mdpi.com/article/10.3390/medicina62061194/s1, Table S1: Normative data for handgrip strength by age and sex based on Mathiowetz et al. reports, together with the 20th percentile.

## Abbreviations

The following abbreviations are used in this manuscript:

IBD	Inflammatory bowel disease
CD	Crohn’s disease
UC	Ulcerative colitis
CDAI	Crohn’s Disease Activity Index
NHANES	National Health and Nutrition Examination Survey
CES-D	Center for Epidemiologic Studies Depression
HGS	Handgrip strength
IPAQ	International Physical Activity Questionnaire
EWGSOP	European Working Group on Sarcopenia in Older People
MAMC	Mid-arm muscle circumference
MUAC	Mid-upper arm circumference
TSF	Triceps skinfold thickness
BMI	Body mass index
OR	Odds Ratio
