# Supplementary material for: Factors Associated with Frailty in Patients with Active Inflammatory Bowel Disease: A Single-Center Observational Study
Source: Medicina (Kaunas). 2026 Jun 21;62(6):1194. doi: 10.3390/medicina62061194 (PMC13302993; doi:10.3390/medicina62061194)
Supplement: Supplementary file 1 [file medicina-62-01194-s001.zip › medicina-4334800-supplementary.pdf]

Supplementary Material

Factors Associated with Frailty in Patients with Active Inflammatory Bowel Disease: A Single-Center Observational Study

Authors: Mihaela Topala [1,2*], Victor Ionescu [3,4], Ioana Gabriela Lupescu [5,6], Gabriel Becheanu [7,8] and Cristian Gheorghe [2,9]

Affiliations: [1]Doctoral School, University of Medicine and Pharmacy "Carol Davila", Bucharest, Romania; [2]Center of Gastroenterology and Hepatology, Fundeni Clinical Institute, Bucharest, Romania; [3]Infection Control Unit, Bucharest Clinical Emergency Hospital, Bucharest, Romania; [4]Center for Excellence in Translational Medicine, Fundeni Clinical Institute, Bucharest, Romania; [5]Department of Radiology, Medical Imaging and Interventional Radiology I, University of Medicine and Pharmacy "Carol Davila", Bucharest, Romania; [6]Department of Radiology and Medical Imaging, Fundeni Clinical Institute, Bucharest, Romania; [7]Department of Pathology, University of Medicine and Pharmacy "Carol Davila", Bucharest, Romania; [8]Department of Pathology, Fundeni Clinical Institute, Bucharest, Romania; [9]Discipline of Gastroenterology and Hepatology, Department of Internal Medicine, University of Medicine and Pharmacy "Carol Davila", Bucharest, Romania; *Correspondence: elena-mihaela.topala@drd.umfcd.ro; Tel.: +40721337279

Table S1. Normative data for handgrip strength by age and sex based on Mathiowetz et al reports, together with the 20th percentile

		MALES			FEMALES		
**AGE**	**HAND**	**MEAN**	**SD**	**P20**	**MEAN**	**SD**	**P20**
18-19	R	49.00	11.20	39.59	32.50	5.60	27.78
	L	42.20	12.60	31.62	28.00	5.70	23.21
20-24	R	54.90	9.30	47.09	31.90	6.60	26.36
	L	47.40	9.90	39.08	27.70	5.90	22.74
25-29	R	54.80	10.40	46.06	33.80	6.30	28.51
	L	50.10	7.30	43.97	28.80	5.50	24.18
30-34	R	55.20	10.20	46.63	35.70	8.70	28.39
	L	50.10	9.80	41.87	30.80	8.00	24.08
35-39	R	54.30	10.90	45.14	33.60	4.90	29.48
	L	51.20	9.80	42.97	30.10	5.30	25.65
40-44	R	53.00	9.40	45.10	31.90	6.10	26.78
	L	51.20	8.50	44.06	28.30	6.30	23.01
45-49	R	49.80	10.40	41.06	28.20	6.80	22.49
	L	45.70	10.30	37.05	25.40	5.80	20.53
50-54	R	51.50	8.20	44.61	29.80	5.30	25.35
	L	46.20	7.70	39.73	26.00	4.90	21.88
55-59	R	45.90	12.10	35.74	26.00	5.70	21.21
	L	37.70	10.60	28.80	21.50	5.40	16.96
60-64	R	40.70	9.30	32.89	25.00	4.60	21.14
	L	34.80	9.20	27.07	20.70	4.60	16.84
65-69	R	41.30	9.30	33.49	22.50	4.40	18.80
	L	34.80	9.00	27.24	18.60	3.70	15.49
70-74	R	34.20	9.80	25.97	22.50	5.30	18.05
	L	29.40	8.20	22.51	18.80	4.60	14.94

Abbreviations: L – left; P20 – percentile 20; R – right; SD – standard deviation

Mathiowetz, V.; Kashman, N.; Volland, G.; Weber, K.; Dowe, M.; Rogers, S. Grip and pinch strength: normative data for adults. *Arch Phys Med Rehabil*. **1985**, *66(2)*: 69-74.
